# The functional evaluation of *Pichia pastoris* hydrolysate as a protein source partial replacement of soybean meal in diets of growing-finishing pigs

**DOI:** 10.1186/s40813-026-00511-7

**Published:** 2026-04-17

**Authors:** Chunyan Xie, Ning Li, Hongwei Liu, Shiyue Liang, Lumin Gao, Zhengqun Liu, Shuqin Mu, Shuguang Liu, Xin Wu

**Affiliations:** 1 Tianjin Key Laboratory of Animal Molecular Breeding and Biotechnology, Tianjin Livestock and Poultry Health Breeding Technology Engineering Center, Institute of Animal Science and Veterinary, Tianjin Academy of Agricultural Sciences, Tianjin, 300381 People’s Republic of China; 2 Tianjin Institute of Industrial Biotechnology, Chinese Academy of Sciences, Tianjin, 300308 China; 3 BeiJing Chase Future Biotech Co., Ltd., Beijing, 102600 China

**Keywords:** *Pichia pastoris* hydrolysate, Growth performance, Carcass traits, Immune status, Gut microbiome

## Abstract

**Background:**

As the most important plant-based protein ingredient in animal husbandry, soybean meal has become key focu in the feed industrym highlighting the need to reduce its inclusion rate and identify alternative protein sources. *Pichia pastoris* hydrolysate (PPH) has regarded as an ideal single-cell protein (SCP) due to its efficient and low-cost protein biosynthesis. This study aimed to evaluate the effects of PPH partial substitution of soybean on the growth performance, immune status, gut microbe and meat quality of growing-finishing pigs.

**Methods:**

A total of 180 pigs with similar weight (31.48 ± 0.91 kg) were divided into 5 groups, including the control group (fed with basal diet) and treatment groups with PPH levels of 1.25%, 2.5%, 3.75% and 5%, respectively. Every group had 6 replicates, and each replicate consisted of 6 pigs. After 76 days on feed, all pigs were weighted, then one pig/replicate was slaughtered to collect blood samples and fresh fecal samples for further analysis, as well as evaluating the carcass traits and meat quality of pigs.

**Results:**

PPH supplement improved the growth performance and carcass traits of pigs, especially the 2.5% PPH replacing 14.18% soybean meal, although there was no obvious difference in meat quality between control group and 2.5% PPH group. Results of serum immune-related factors showed that PPH supplement markedly elevated antioxidant status, increased serum anti-inflammatory factors and immunoglobulin levels, and strengthened the intestinal barrier of pigs. Fecal microbiome sequencing indicated that PPH could modulate fecal microbiome diversity, and promoted the probiotics proportion in the gut of pigs to varying degrees.

**Conclusions:**

Above findings revealed that partial replacement of soybean with PPH, not only significantly improved the growth performance and carcass traits of growing-finishing pigs, but also immunity performance and intestinal microecology, which provide insights into the application possibility of the PPH serving as an alternative feed ingredient in swine production system to offer excellent protein sources.

**Supplementary Information:**

The online version contains supplementary material available at 10.1186/s40813-026-00511-7.

## Introduction

As the primary protein source in commercial livestock diets, soybean meal contributeds to intensified crop production and places substatial pressure on water and agricultural land resources [1, 2]. Further, the global supply of soybean meal is insufficient to meet rapidly growing demands of the animal husbandry industry [3]. Thus, there is an urgent need to identify more sustainable, accessible, and health-promoting protein sources that can partially or fully replace conventional protein ingredients, especially in China. Driven by increasing market demand for healthier animal-derived products, the livestock sector is under pressure to adopt natural and residue-free feed additives [4]. Microbial protein from bacteria and fungi has emerged as a promising alternative due to its lower land and water requirements, as well as less carbon emissions [5]. Particularly, yeast provides essential amino acids, trace minerals, and vitamins [6], and has been used in food and feed for decades without anti-nutritive ingredients or a beany taste [7–10]. Studies showed that dietary yeast or yeast hydrolysate could enhance growth performance, digestion and immunity in livestock [11, 12]. Besides protein, polysaccharides, nucleotides and some growth-related factors in yeast also exhibit immunomodulatory and antioxidant effects [13]. Our previous study indicated that yeast-based nucleotide improved intestinal barrier function and immunity in neonatal piglets [14]. Certainly, hydrolyzed yeast can be a more cost-effective alternative than yeast extracts.

Despite the identification of over a thousand yeast strains, only few, such as *Saccharomyces cerevisiae* or *Saccharomyces boulardii*, are widely used in animal feed, primarily due to cost and efficacy considerations [15, 16]. *Pichia pastoris* is already recognized as an effective platform for recombinant protein production, and has shown benefits for growth performance and immunity in piglets [17, 18]. Regrettably, limited data are available on the use of *P. pastoris* or its hydrolysate as feed additive. In this study, the mild strain *P. pastoris* C1 was considered as an ideal SCP biosynthesis platform based on one-carbon (C1) compounds, as it can be produced not only from low-quality coal-derived substrates, but also from hydrogen and carbon dioxide (CO_2_) [19], making it a promising sustainable and economical protein source in animal feed. Nevertheless, evidences regarding the application of wild *P. pastoris* or its hydrolysate in swine remains scarce. Thus, this study aimed to evaluate the feeding value of PPH as partial substitution of soybean meal in the diet via growth performance, carcass traits, immune status and gut microbial diversity of growing-finishing pigs, then diversify the protein source for animal feeds.

## Materials and methods

### Preparation of PPH

*Pichia pastoris* C1 strain was isolated and domesticated by Tianjin Institute of Industrial Biotechnology, Chinese Academy of Sciences. PPH used in this study contains 50.49% crude protein, polysaccharides (8.8%), phosphorus (1.73%), calcium (0.03%), and nucleic acids (0.2%), which was produced by BeiJing Chase Future Biotech Co. Ltd., China. The preparation detail of PPH was according to the description in the study of Jiao Meng, et al. [19]. 

### Animal design and diets

This trial was conducted at the Tianjin Modern Animal Husbandry Innovation Demonstration Base, an efficacy evaluation base for new feedstuffs authorized by the Ministry of Agriculture and Rural Affairs. In addition, this experiment was approved by the Ethics and Animal Welfare Committee of Tianjin Academy of Agricultural Sciences (approval No: 2024001).

A total of 180 healthy “Landrace × Yorkshire” dual-cross pigs with similar parity, age and weight (31.48 ± 0.91 kg) were divided into 5 groups, including the control group without PPH (0, the corn-soybean based diet), and four treatment groups with PPH levels of 1.25%, 2.5%, 3.75% or 5%, respectively. Correspondingly, experiment diets were formulated by replacing 7.14%, 14.18%, 21.46% or 28.51% soybean meal with PPH, respectively. Every group had 6 replicates, and each replicate (one pen) was ensured to contain 6 pigs (male: female = 4:2).

The experiment diets were formulated according to the NRC (2012) and exceeded this standard of nutrient requirements. PPH was included in the diets by replacing part of soybean meal in the basal diet. All diet were produced by Tianjin Yuan Ding Feed Co., Ltd., China. Diet samples were then collected, pulverized and stored at -20 °C for further analysis. The major ingredients and nutrient level of diets are shown in Table 1.

**Table 1:** Ingredients and nutrient level of the pig diets

Item	Control	PPH Diet			
	0	1.25%	2.5%	3.75%	5%
Major ingredients, %					
Corn	69.77	70.04	70.28	70.46	70.69
**Soybean meal**,** 43% CP**	**21.29**	**19.77**	**18.27**	**16.72**	**15.22**
**PPH**	**0.00**	**1.25**	**2.50**	**3.75**	**5.00**
Bran	3.98	3.98	3.98	3.98	3.98
Soybean oil	1.00	1.00	1.00	1.00	1.00
Stone powder	1.39	1.39	1.39	1.39	1.39
CaHPO_4_	1.09	1.09	1.09	1.19	1.19
NaCl	0.30	0.30	0.30	0.30	0.30
L- Lysine sulfate, 70%	0.60	0.60	0.60	0.61	0.62
DL- Methionine, 98.5%	0.05	0.05	0.05	0.05	0.05
L- Threonine, 99.0%	0.05	0.04	0.04	0.04	0.05
L- Tryptophan	0.01	0.02	0.03	0.04	0.04
Premix ^1^	0.34	0.34	0.34	0.34	0.34
Phytase, 1*10^4^ IU/kg	0.04	0.04	0.04	0.04	0.04
Choline chloride, 60%	0.08	0.08	0.08	0.08	0.08
Antioxidants	0.01	0.01	0.01	0.01	0.01
Total	100.00	100.00	100.00	100.00	100.00
Nutrient level ^2^					
Dry matter, %	88.82	88.77	88.98	89.04	88.90
DE, MJ/kg	14.31	14.31	14.31	14.31	14.31
CP, %	16.05	16.24	15.42	15.36	15.28
Crude fat, %	2.48	2.53	2.28	2.51	2.54
Crude fibre, %	2.99	2.97	2.80	2.65	2.61
Ca, %	0.91	0.72	0.87	0.90	0.93
Total P, %	0.62	0.57	0.68	0.66	0.70
Lys, %	1.16	1.15	1.16	1.15	1.16

PPH = *Pichia pastoris* hydrolysate, DE = Digestive energy, CP = Crude protein

^1^ The premix provided the following per kilogram of diet: VA 6500 IU, VD_3_ 2400 IU, VE 20 mg, VK_3_ 2.4 mg, VB_1_ 2.4 mg, VB_2_ 6.6 mg, VB_6_3 mg, VB_12_ 0.025 mg, niacin 25 mg, pantothenic acid 13 mg, biotin 0.2 mg, Cu 25 mg, Fe 150 mg, Zn 80 mg, Mn 50 mg, I 0.6 mg, Se 0.3 mg

^2^ Nutrient levels are measured values except for DE

Before the animal trial started, diets were gradually transitioned from the basic diet to the treatment diet during the pre-feeding period. Specifically, on the 1st, 3rd, and 5th days of this period, pigs received 10%, 50%, and 100% of the experimental diet, respectively. The animal trail lasted for 76 days after 5 days pre-feeding. All the pigs had free access to water and feed, and the ambient temperature was kept between 20 °C and 28 °C. Further, the feed intake and health status of pigs were recorded daily.

Two pigs, one from the 3.75% group and one from the 5% PPH group, were removed due to paralysis during the trial, and their relevant data were excluded from the final growth performance analysis.

### The growth performance of pigs

The weight of pigs at the start and end of the trial, along with daily feed intake of each replicate were recorded to calculate the average daily gain (ADG) and average daily feed intake (ADFI) of pigs. Then, the feed/gain (F/G) of pigs was calculated at the end of the trial.

### Sampling

One male pig/replicate, with body weight close to the average, was selected for blood sampling from the sinus of the anterior vena cava after 12 h of fasting. Serum samples were obtained after being centrifuged at 3000 *g* for 20 min at 4 °C for detecting serum biochemical parameters, immune-related indicators and antioxidant indices. Blood samples with anticoagulant were prepared for routine blood parameters. Fecal samples were collected from the deep part of fresh feces and immediately frozen in liquid nitrogen for microbial gene sequencing.

### Determination of routine blood parameters and serum biochemical parameters

Anticoagulated blood samples were tested for routine blood parameters using an automatic blood cell analyzer (Mindray 5180 CRP, Ruipu Medical equipment Co., LTD, Changchun, China).

Serum biochemical parameters, including total protein (TP, Cat. 03183734190), albumin (ALB, Cat. 03183688122), ammonia (Amm, Cat. 20766682322), alanine aminotransferase (ALT, Cat. 20764957322), aspartate amino transferase (AST, Cat. 20764949322), alkaline phosphatase (ALP, Cat. 03333701190), blood urea nitrogen (BUN, Cat. 04460715190), creatinine (CREA, Cat. 04810716190), triglyceride (TG, Cat. 20767107322), total cholesterol (TCHO, Cat. 03039773190), low-density lipoprotein (LDL, Cat. 03038866322) and high-density lipoprotein (HDL, Cat. 04399803190) were measured with commercial kits (Roche Diagnostics GmbH, German) using an automatic biochemical analyzer (Cobas c311, Roche Diagnostics, Switzerland).

### Detection of serum immunological mediators/antioxidant-related indices

The serum antioxidant-related indices, including superoxide dismutase (SOD, Cat. A001-1-2), glutathione peroxidase (GSH-Px, Cat. A005-1-2), total antioxidant capacity (T-AOC, Cat. A015-1-2), malondialdehyde (MDA, Cat. A003-1-2) were detected using colorimetry method by kits purchased from Nanjing Jiancheng Bioengineering Institute (Nanjing, China); The serum immunological mediators, such as serum interleukin-2 (IL-2, Cat. 88-7025), interleukin-4 (IL-4, Cat. 88-7046), interleukin-6 (IL-6, Cat. 88-7066), interleukin-10 (IL-10, Cat. 88–710), tumor necrosis factor-α (TNF-α, Cat. 88-7346), interferon gamma (IFN-γ, Cat. 88-7316) were determined using the enzyme-linked immunosorbent assay (ELISA) kits from Beijing CapitalBio Corporation, and serum immunoglobulin G (IgG, Cat. JH-00013), immunoglobulin M (IgM, Cat. JH-00015), immunoglobulin A (IgA, Cat. JH-00014) were determined using the ELISA kits from Beijing Jinhai Keyu Biotechnology Development Co., Ltd. All the tests were carried out according to the instructions.

Additionally, as key indicators of intestinal permeability, both serum diamine oxidase (DAO, Cat. A088-1-1, Nanjing Jiancheng Bioengineering Institute, China) and D-lactic acid (D-LA, Cat. G0827F, Grace Biotechnology, Suzhou, China) were also determined to assess the intestinal barrier integrity of growing-finishing pigs.

### Fecal microbial sequencing

According to the sampling requirements for microbial sequencing, fresh pig feces samples (one pig/replicate) were collected on the morning of day 76 of the experiment after fasting overnight. About 2 g of fecal samples at a depth of 3–5 cm were collected using a sterile sampling spoon and immediately transferred into liquid nitrogen and − 80 °C for fecal microbiota analysis.

The total DNA of the fecal samples was extracted using the MagPure Stool DNA KF Kit (Magen Biotechnology Co., Ltd., Guangzhou, China). Then, amount and quality of the extracted microbial DNA were detected by 2% agarose gel electrophoresis and a UV spectrophotometer (Thermo Fisher Scientific, Waltham, MA, USA), respectively. Specific primers targeting the V3-V4 hypervariable region of the bacterial 16 S rDNA gene were: 338 F (5’-ACTCCTACGGGAGGCAGCAG-3’) and 806R (5’-GGACTACHVGGGTWTCTAAT-3’). PCR amplification was performed using an ABI GeneAmp^®^9700 PCR instrument (Applied Biosystems, Foster City, CA, USA) with a 20 µL reaction system containing TransStart Fastpfu DNA Polymerase (TransGen AP221-02). Sequencing and data analysis were conducted by Shanghai Yuanshen Biotechnology Co., Ltd. (Shanghai, China) using the Illumina NovaSeq 6000 PE250 (Illumina Inc., San Diego, CA, USA). The resulting sequences were clustered into OTUs (Operational Taxonomic Units) at a similarity level of 97% and subjected to bioinformatics statistical analysis using software Mothur (version v.1.30.1) and Usearch (version 7.0).

### Slaughter, carcass traits & meat quality

Based on growth performance, one pig/replicate in the control group and 2.5% PPH group was electrically stunned, exsanguinated, decapitated, dehaired, and split down the midline according to standard commercial procedure. Then, fresh longissimus thoracis (LT) samples anterior to the 13th rib from the left side carcass were collected for the meat characteristics evaluation and meat physical analysis after the left half was weighed. Meanwhile, about 20 g LT sample was collected and stored at -20 °C for subsequent free amino acid and fatty acid analysis. The carcass traits were evaluated according to a previous study [20].

#### Determination of carcass traits

Within 30 min postslaughter, hot carcass weight was recorded to calculate the carcass yield using the following formula: carcass yield = (carcass weight/live weight) × 100%. The carcass straight length was measured by a tape measure from the anterior edge of the base of the atlanto-occipital joint to the anterior edge of the pubic symphysis, and the carcass oblique length was measured from the junction of the first rib and the sternum to the midline of the pubic symphysis. The backfat thickness was defined by Vernier caliper at the 13th rib, then the left side carcass was cut between the 12th rib and the 13th rib to determine the loin eye area using the following formula: length × width × 0.7.

#### Determination of meat characteristics of LT

Subjective meat characteristics of LT were evaluated as described previously [21]. The pH of LT muscle at 45 min and 24 h post mortem was measured with a portable pH meter (Hanna Instruments, Cluj L Napoca, Romania). The color of LT muscle 2 h post mortem was measured using a Colorimeter (CR-300, Minolta Co. Ltd., Osaka, Japan), and the lightness (L*), Redness (a*), Yellowness (b*) values were determined in triplicate. The pressure plate of the instrument (Stable Micro Systems, ta.xtplus, Britain) and texture analyzer (TA.XT Plus, Stable Micro Systems, Godalming, UK) were used to determine the pressure loss and shear force of LT, respectively. Moreover, subjective muscle marbling score was determined using the National Pork Producer Council standards (National Pork Producers Council, 1999).

#### Free amino acids (FAAs) analysis

Approximately 0.5 g freeze-dried LT sample was weighed and homogenized with 4 mL 0.01 N hydrochloric acid to extract with ultrasonic for 30 min. Then, 2 mL extracted supernatant and 2 mL of n-hexane were mixed for lipid removal. After the layers separated, 1 mL of the lower layer was taken and mixed with 1 mL of 8% sulfosalicylic acid and left to stand at 4 °C for at least 30 min. The mixture was then centrifuged at 10,000 rpm for 10 min at 4 °C. Finally, the supernatant was filtered through a 0.22 μm membrane, and FAAs were detected using an amino acid analyzer (L8900, Hitachi, Japan).

#### Long-chain fatty acids (LCFAs) analysis

After taking 0.5 g freeze-dried LT sample to 5 mL mixture (benzene: petroleum ether = 1:1) for 24 h sealed extraction, about 5 mL of 0.4 mol/L sodium hydroxide methanol solution was added to vortex and stand for 30 min. Next, adding 3 mL of ultrapure water and centrifuge at 3000 r/min for 3 min, the upper solution was transferred to a tube containing 3 g of anhydrous sodium sulfate to stand for 5 min to filter through 0.22 μm membrane. Finally, the upper solution was diluted 5-fold with n-hexane, and then LCFAs were detected with a gas chromatograph (7890 A, Agilent Technologies, USA).

### Statistical analysis

In the animal trial, the concentration of PPH in the diet was specified as a fixed effect, the growth performance, routine blood parameters, serum biochemical indices, serum immune-related factors and intestinal permeability markers, composition of fecal microbes, carcass traits, FAAs and LCFAs profiles were specified as random effects. Data were analyzed using GraphPad Prism version 9.5.0 (GraphPad Software, USA) and results presented as the mean + standard error of the mean (SEM) or total SEM. The statistical significance between the PPH group and control group is indicated as *P ≤ 0.05, **P ≤ 0.01 and ***P ≤ 0.001.

The growth performance, routine blood parameters, serum biochemical indices, serum immune-related factors and intestinal permeability markers, composition of fecal microbes were analyzed by one-way ANOVA with Duncan multiple-range test and LSD. Carcass traits, meat characteristics, FAAs and LCFAs of LT muscle were analyzed by independent-samples *t*-test. *P* ≤ 0.05 was considered statistically significant among different groups. Besides, the quadratic regression analysis of final weight and ADG were made to evaluate the linear or quadratic effects between the PPH levels and the growth performance of pigs. The *P* value less than 0.05 means a significant linear effect or quadratic effect between the PPH level and the index of growth performance.

Finally, the Pearson correlation analysis was performed to evaluate the relationships among intestinal barrier markers, serum cytokines and fecal microbe at the genus level.

## Results

### Effect of dietary level of PPH on the growth performance of pigs

The effect of PPH supplement on the growth performance of pigs is shown in Table 2. Compared with the control group, pigs fed with 1.25%, 2.5%, 3.75% and 5% PPH, increased by 0.9 kg, 6.1 kg, 4.3 kg, and 1.3 kg in the final weight (*P* < 0.05), respectively, and increased by 0.017 kg, 0.082 kg, 0.056 kg and 0.015 kg in the ADG (*P* < 0.05), respectively. Correspondingly, the ADFI increased by 0.08 kg, 0.243 kg, 0.132 kg, and 0.106 kg, respectively. Most notably, the final weight (*P* < 0.05) and ADG (*P* < 0.05) of pigs from 2.5% PPH group had obvious improvement when compared with those in the control group, 1.25% group and 5% group, although no statistic difference (*P* > 0.1) was observed in the ratio of F/G and ADFI among five groups.

**Table 2:** Effect of PPH supplement on the growth performance of pigs

Item^1^	Control	PPH Diet				SEM	*P*- Value	*P*-Value	
	0	1.25%	2.5%	3.75%	5%			Linear	Quadratic
Initial weight, kg	31.61	31.20	31.42	31.61	31.79	0.163	0.846	0.515	0.575
Final weight, kg	111.7b	112.6b	117.8a	116.0ab	113.0b	0.768	**0.048**	0.270	**0.033**
ADG, kg	1.054b	1.071b	1.136a	1.110ab	1.069b	0.010	**0.028**	0.318	**0.016**
ADFI, kg	2.949	3.029	3.192	3.081	3.055	0.031	0.183	0.225	0.088
F/G	2.812	2.829	2.741	2.864	2.858	0.029	0.703	0.543	0.670

PPH = *Pichia pastoris* hydrolysate; ADG = Average daily gain; ADFI = Average daily feed intake; F/G = Feed/Gain

^1^ Values are expressed by means with total SEM, *n* = 6. Values within columns with different lowercase (s) indicate significant differences (*P* ≤ 0.05)

**Fig. 1:**
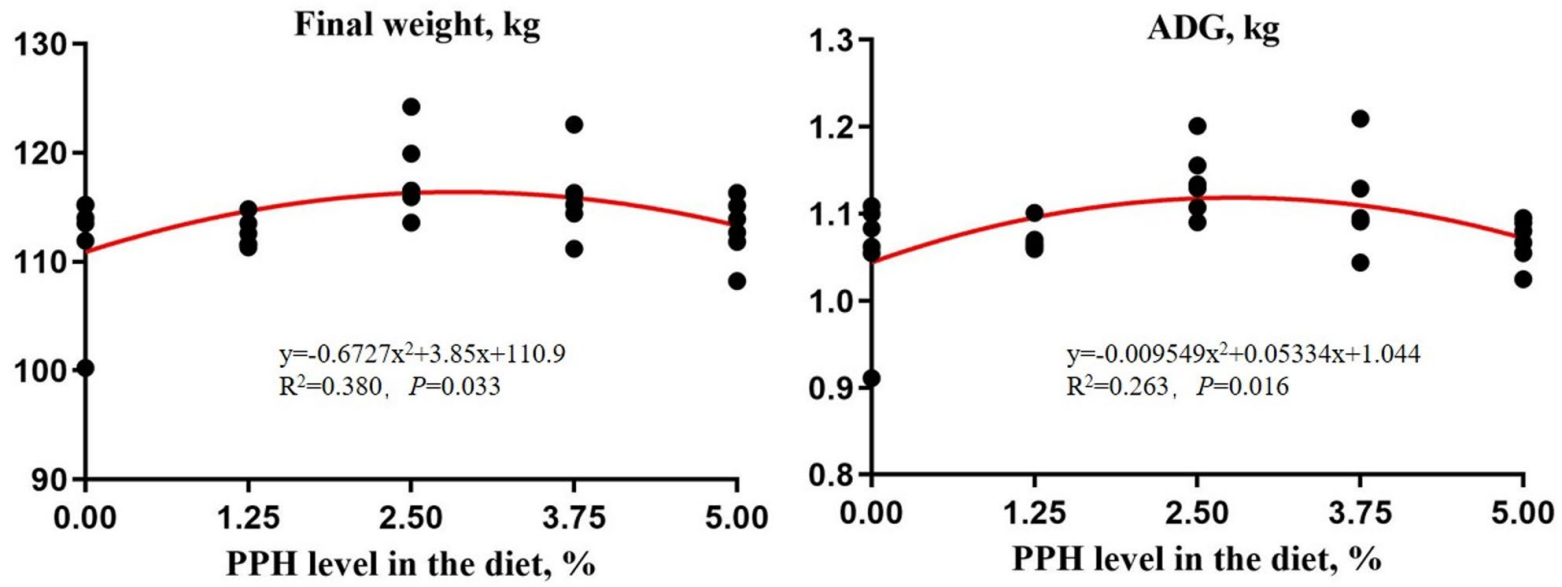
The quadratic regression analysis of final weight and ADG. Values are expressed by the means, *n* = 6. PPH = *Pichia pastoris* hydrolysate; ADG = Average daily gain

The PPH level had significant quadratic effects on the final weight (*P* < 0.05) and ADG (*P* < 0.05) of growing-finishing pigs (Table 2). Quadratic regression analysis showed that the final weight of pigs maximized when the addition level of PPH was to 2.86%, and the ADG reached the peak when the proportion of PPH in the diet was 2.79%, indicating that pigs fed diet with 2.79% PPH would maximize the production efficiency of growing-finishing pigs (Fig. 1).

### The immune-related cells in the blood of growing-finishing pigs

Results of routine blood parameters showed that 5% PPH supplement increased (*P* < 0.05) blood eosinophils, basophils and their proportions of pigs when compared with those in the control group, although there was no statistical difference among different PPH groups. There is no obvious difference in other routine blood parameters between PPH groups and control group (Table 3, and the complete result of routine blood parameters is presented in the additional file 1).

**Table 3:** Effect of PPH supplement on the routine blood parameters of growing-finishing pigs

Item^1^	Control	PPH Diet				SEM	*P*- Value
	0	1.25%	2.5%	3.75%	5%		
WBC, 10^9^/L	18.26	19.51	19.47	15.60	21.56	1.342	0.742
Neutrophils, 10^9^/L	1.867	1.935	1.707	1.563	3.017	0.197	0.135
Lymphocytes, 10^9^/L	16.14	17.31	17.49	13.81	18.03	1.219	0.844
Monocytes, 10^9^/L	0.178	0.138	0.183	0.118	0.212	0.018	0.481
Eosinophils, 10^9^/L	0.078	0.115	0.087	0.088	0.228*	0.019	0.062
Basophils, 10^9^/L	0.002	0.017	0.000	0.017	0.067*	0.009	0.147
Neutrophils, %	9.955	9.560	8.835	13.617	14.433	1.088	0.369
Lymphocytes, %	88.62	89.14	89.75	84.12	83.33	1.304	0.384
Monocytes, %	0.958	0.718	0.965	1.483	0.933	0.179	0.764
Eosinophils, %	0.452	0.532	0.447	0.700	1.083*	0.092	0.145
Basophils, %	0.017	0.050	0.000	0.083	0.217*	0.027	0.074

^1^ Values are expressed by means with total SEM, *n* = 6. * Means 0.01 < *P* ≤ 0.05 between control group and PPH group. WBC = White blood cell

### The serum biochemical indices of growing-finishing pigs

As shown in Table 4, compared with the control group, serum ALB of pigs (*P* < 0.05) fed with PPH diet increased to different extents, especially the 2.5% PPH group (*P* < 0.01). In the absence of obvious disease throughout the whole trial, serum CREA also pretended the similar tendency in the 1.25% (*P* < 0.01) and 5% (*P* < 0.01) PPH groups. It is worth noting that PPH supplement significantly reduced (*P* < 0.001) serum Amm of pigs, especially the 5% PPH level when compared with those in the control group and other PPH groups. In comparison, the serum lipid (TG, TCHO, LDL, HDL), TP, ALT, AST, ALP, and BUN didn’t differ obviously among five groups (*P* > 0.1).

**Table 4:** Effect of PPH supplement on the serum biochemical indices of growing-finishing pigs

Item^1^	Control	PPH Diet				SEM	*P*- Value	
	0	1.25%	2.5%	3.75%	5%			
TP, g/L	71.48	70.40	71.27	70.63	71.92	0.540		0.915
ALB, g/L	50.23c	53.68ab	54.37a	51.05bc	53.38ab	0.521		**0.035**
ALT, U/L	64.23	58.20	61.07	58.67	63.63	1.627		0.707
AST, U/L	35.17	37.17	40.50	38.00	36.17	1.068		0.589
ALP, U/L	183.7	186.0	190.3	216.8	209.7	6.245		0.345
BUN, mmol/L	5.72	5.58	5.70	5.17	5.82	0.180		0.831
Amm, µmol/L	270.5a	238.3b	247.4ab	238.7b	207.1c	5.219		**< 0.001**
CREA, µmol/L	97.33b	120.0a	109.3ab	110.8ab	120.7a	2.756		**0.035**
TG, mmol/L	0.51	0.46	0.56	0.51	0.50	0.019		0.510
TCHO, mmol/L	2.65	2.58	2.67	2.75	2.54	0.049		0.713
LDL, mmol/L	1.45	1.47	1.48	1.48	1.34	0.031		0.601
HDL, mmol/L	1.13	1.06	1.10	1.22	1.14	0.027		0.430

TP = Total protein; Amm = Ammonia; ALB = Albumin; ALT = Alanine aminotransferase; AST = Aspartate amino transferase; ALP = Alkaline phosphatase; BUN = Blood urea nitrogen; Amm = Ammonia; CREA = Creatinine; TG = Triglyceride; TCHO = Total cholesterol; LDL = Low-density lipoprotein; HDL = High-density lipoprotein

^1^ Values are expressed by means with total SEM, *n* = 6. Values within columns with different lowercase (s) indicate significant differences (*P* ≤ 0.05)

### The serum antioxidant-related indicators of growing-finishing pigs

**Fig. 2:**
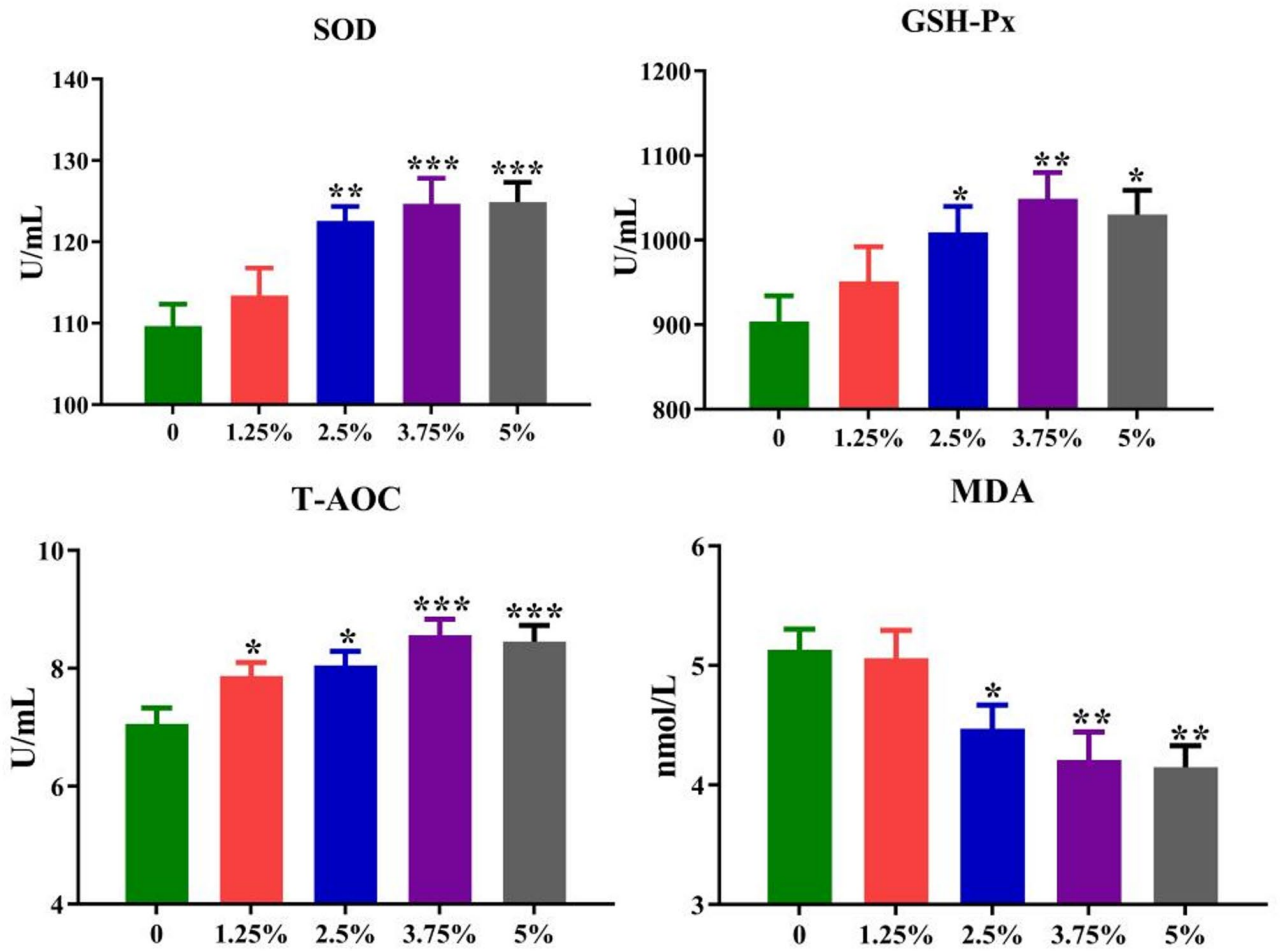
The serum antioxidant-related indicators of growing-finishing pigs. Values are expressed by means + SEM, *n* = 6. The *, **, *** means *P* ≤ 0.05, *P* ≤ 0.01 and *P* ≤ 0.001 between the PPH group and control group, respectively

The serum SOD, GSH-Px, T-AOC and MDA are critical indicators reflecting the antioxidant status. Compared with the control group, the activities of serum SOD (*P* = 0.001), GSH-Px (*P* < 0.05) and T-AOC (*P* < 0.01) were significantly improved to different degrees, while the serum MDA content (*P* < 0.01) was remarkably decreased by providing 2.5%-5% PPH to pigs, suggesting that dietary PPH could improve the antioxidant capacity of pigs (Fig. 2).

### The serum inflammatory-related factors and immunoglobulins of growing-finishing pigs

**Fig. 3:**
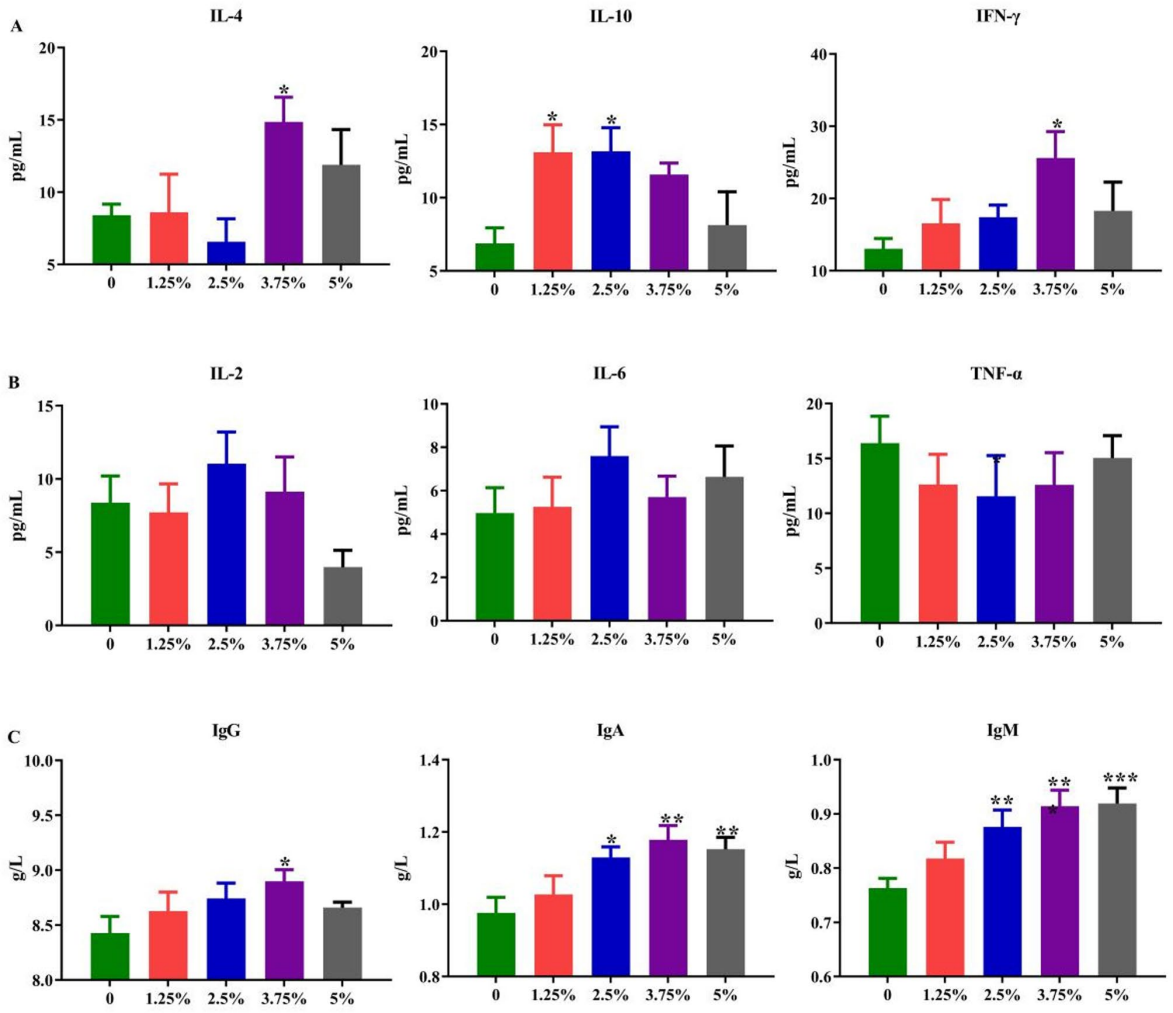
The serum inflammatory-related factors and immunoglobulins of growing-finishing pigs. **A**, serum anti-inflammatory factors. **B**, serum pro-inflammatory factors. **C**, serum immunoglobulin. Values are expressed by means + SEM, *n* = 6. The *, **, *** means *P* ≤ 0.05, *P* ≤ 0.01 and *P* ≤ 0.001 between the PPH group and control group, respectively

Consistently, the serum anti-inflammatory factors (Fig. 3A) and immunoglobulins (Fig. 3C) were significantly improved by PPH in the diet. The 2.5% PPH supplement could improve the serum IgA (*P* < 0.05), IgM (*P* < 0.01) and anti-inflammatory factor IL-10 (*P* < 0.05). Significantly, feeding with 3.75% PPH diet could increase the serum IgG (*P* < 0.05), IgA (*P* < 0.01) and IgM (*P* < 0.001), the anti-inflammatory factor IL-4 (*P* < 0.05) and IFN-γ (*P* < 0.05) of pigs, and the 5% addition also significantly increased the serum IgA (*P* < 0.01) and IgM (*P* < 0.001) when compared with those in the control group, even the serum IL-10 (*P* < 0.05) in the 1.25% group did increase. However, there is no statistical difference in pro-inflammatory factors IL-2, IL-6 and TNF-α among different groups (Fig. 3B). Above results proved that dietary supplement with 1.25%-5% PPH to replace partial soybean could reduce the inflammation level, then improve the immune status of pigs.

### The serum intestinal permeability markers of growing-finishing pigs

**Fig. 4:**
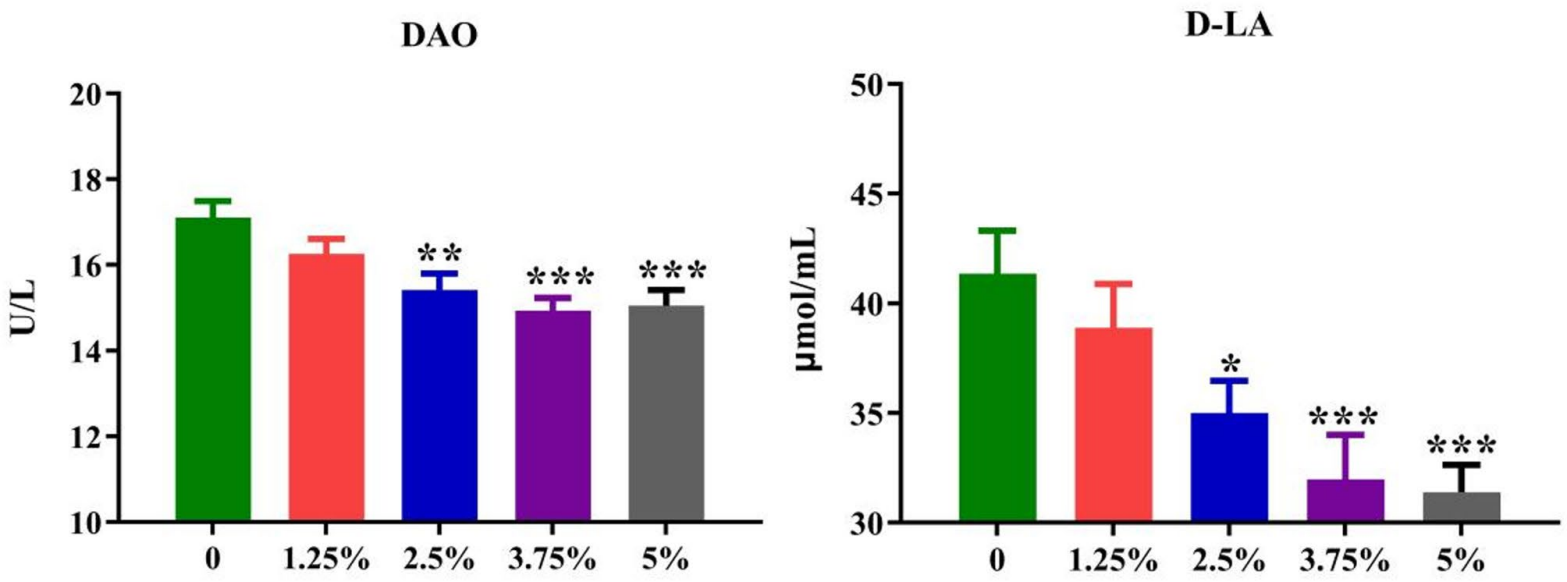
The serum intestinal permeability markers of growing-finishing pigs. Values are expressed by means + SEM, *n* = 6. The *, **, *** means *P* ≤ 0.05, *P* ≤ 0.01 and *P* ≤ 0.001 between the PPH group and control group, respectively

Both serum DAO activity and D-LA content are usually detected to reflect the intestinal permeability, thus mirroring the intestinal barrier of pigs (Fig. 4). In this study, the serum DAO activity (*P* = 0.001) and D-LA concentration (*P* < 0.01) were significantly decreased by PPH supplement when compared with those in the control group, especially when the addition amount of PPH in the diet was between 2.5% and 5%. Above results indicated that there was a negative correlation between the intestinal barrier integrity and the PPH level (≤ 3.75%), indicating that PPH supplement could remarkably reduce the intestinal mucosal permeability, especially the 2.5%-5% level, when replacing 14.18%-28.51% soybean meal in the diet.

### The fecal microbiome composition in growing-finishing pigs

#### Effect of PPH on the alpha (α) diversity of fecal microbiota

**Fig. 5:**
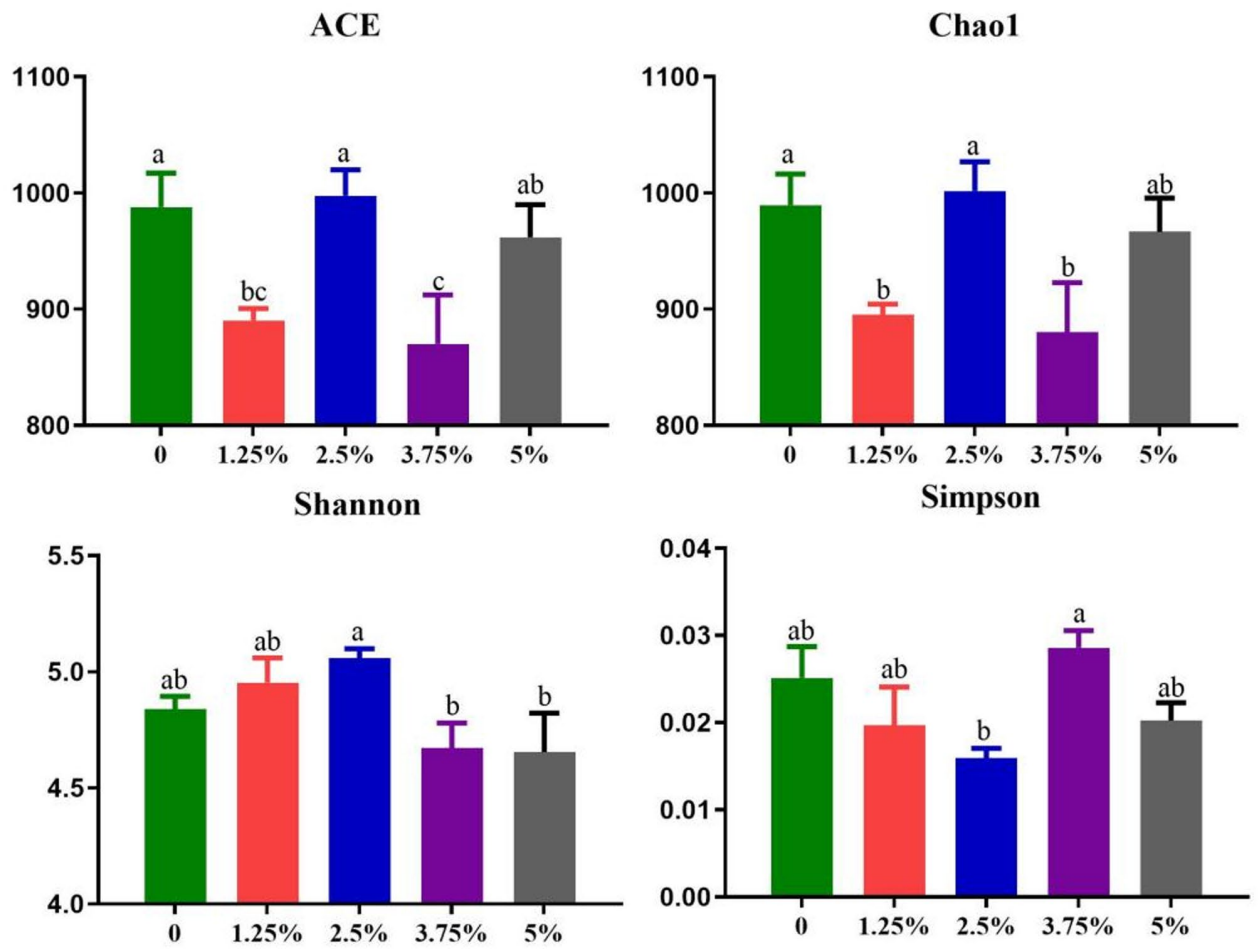
The α diversity of fecal microbiota. Data are presented as mean + SEM, *n* = 6. Values within columns with different lowercases indicate significant differences (*P* ≤ 0.05)

Chao1 and abundance-based coverage estimator (ACE) indices measure microbiota richness, which is the number of species, while Shannon and Simpson indices are used to estimate microbial diversity, which is affected by the abundance and evenness of species in the sample community [22]. As shown in Fig. 5, compared with the control group, the ACE index and Chao1 index of microorganisms in the 1.25% and 3.75% group were lower (*P* < 0.05) than those in the control group, indicating a reduction in the number of microbial species, although 2.5% and 5% had no significant effect on the number of microbial species. The higher the Shannon value, the higher diversity in the community, and Simpson index vice versa. Compared with control group, Simpson index in the 2.5% group significantly reduced (*P* < 0.05), and the Shannon index showed a corresponding increase of 4.76% (*P* < 0.1). Chao1, ACE, Shannon and Simpson indexes indicated that diet with 2.5% PPH could increase the diversity of fecal microbiota without sacrificing the fecal richness of microbial species, which is beneficial to the stability of the intestinal microecological environment. However, there is no obvious difference in the α diversity of fecal microbiota between control group and 5% group.

#### Effects of PPH on the composition of fecal microbes at the phylum and genus levels

**Fig. 6:**
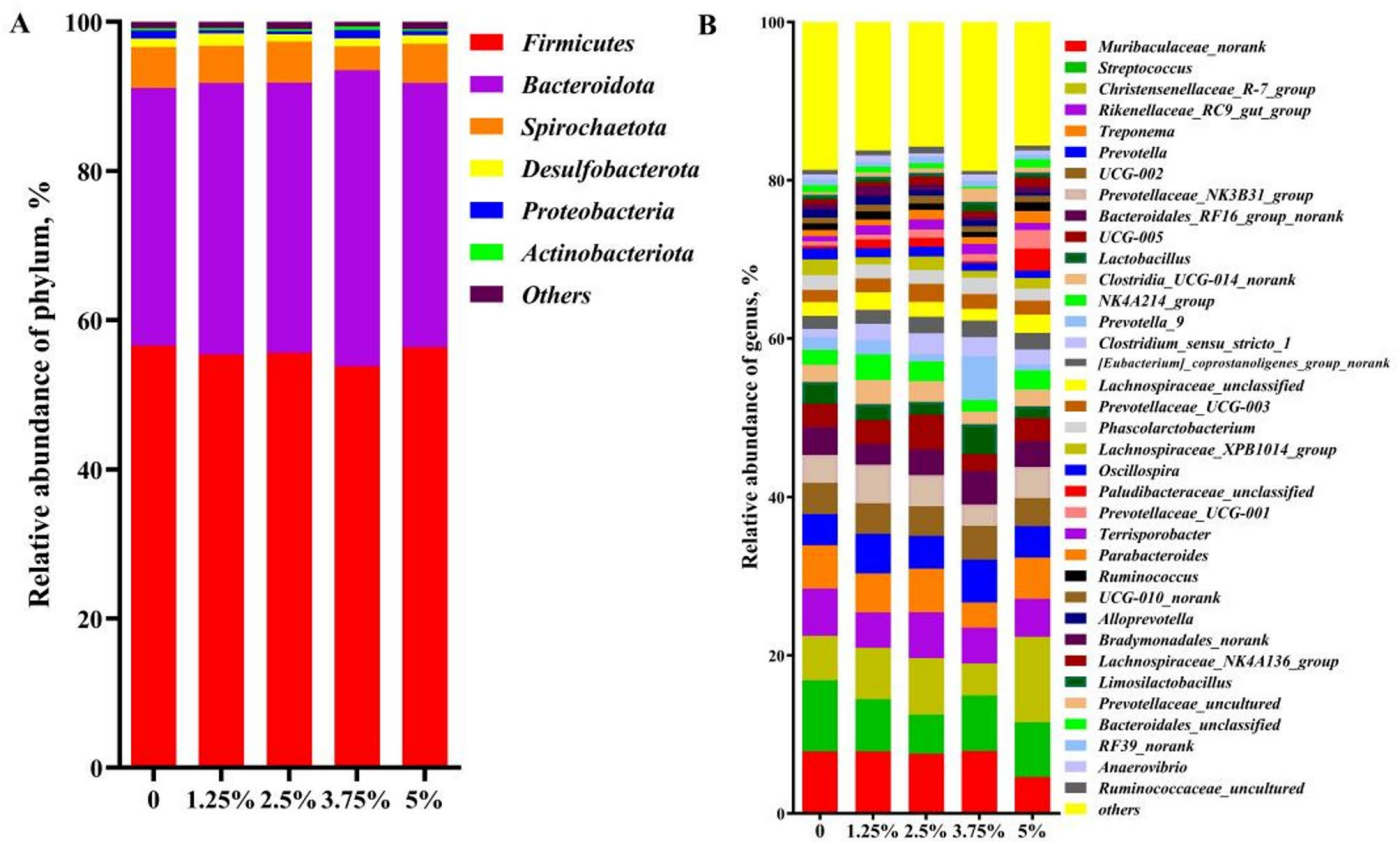
The composition of fecal microbes at the phylum and genus levels. **A**, the phylum level. **B**, the genus level. *n* = 6

As shown in Fig. 6A, the predominant phylum of fecal microbes are *Firmicutes*, *Bacteroidota*, *Proteobacteria*, *Spirochaetota*, *Desulfobacterota* and *Actinobacteriota* et al., which accounted for more than 96% of the abundance of the phylum, and there was no statistically significant difference in bacterial phyla abundances among different diet groups (*P* > 0.1). At the genus level (Fig. 6B), unclassified *Bacteroides* (*Muribaculaceae_norank*), *Streptococcus*, *Christensenellaceae_R-7_group*,* Rikenellaceae_RC9_gut_group*,* Treponema*,* Prevotella*,* Lactobacillus*,* Prevotellaceae_NK3B31_group*, etc. represented the most dominant genera.

#### The relative genus level of fecal microbes with statistical differences 

**Fig. 7:**
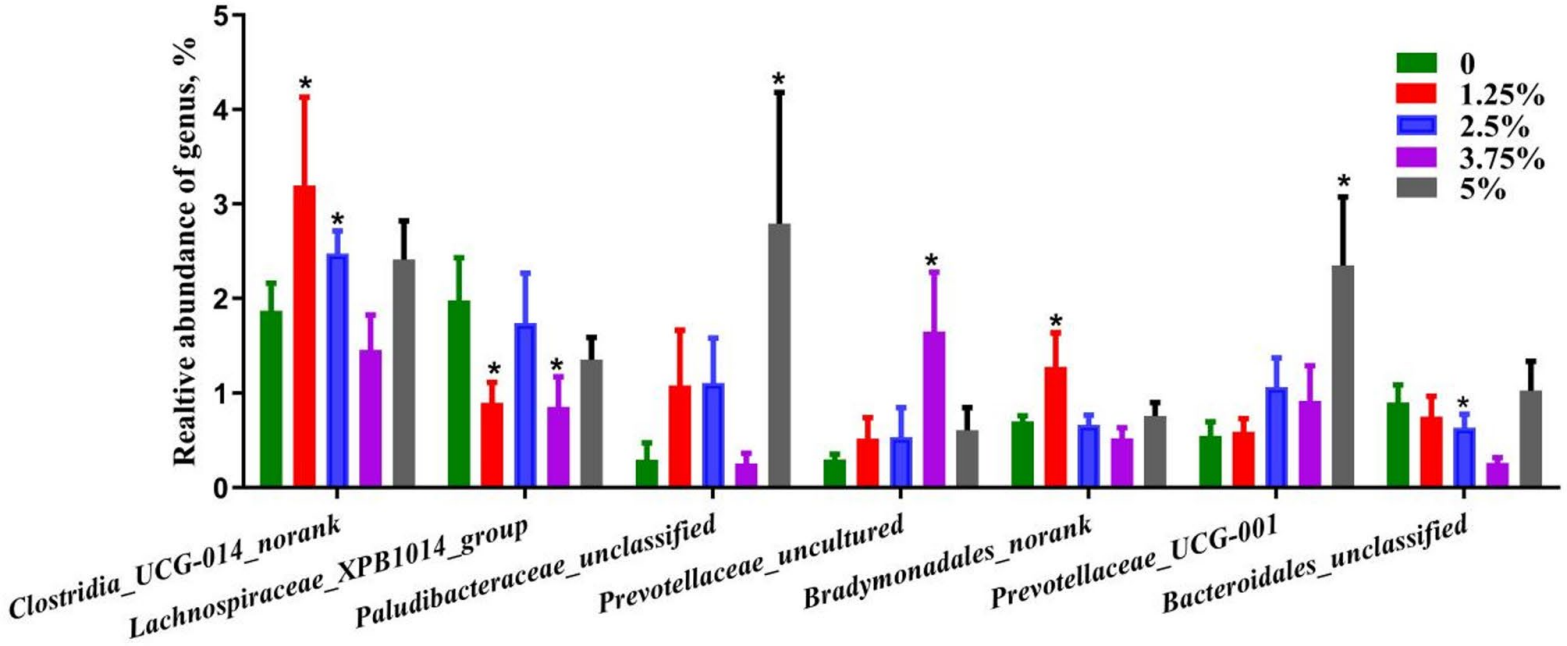
The genus of fecal microbes with statistical difference among five groups. Data are presented as mean + SEM, *n* = 6. * Means 0.01 < *P* ≤ 0.05 between the control group and PPH group

Compared with the control group, the dose of 1.25% PPH increased (*P* < 0.05) the relative abundances of *unclassified Clostridia_UCG-014* and *Bradymonadales*, and 2.5% PPH also increased the relative ratio of *unclassified Clostridia_UCG-014* (*P* < 0.05). The dose of 3.75% PPH increased (*P* < 0.05) the relative abundance of *uncultured Prevotellaceae*, 5% PPH increased the relative abundance of unclassified *Paludibacteraceae* (*P* < 0.05) and *Prevotellaceae_UCG-001* (*P* < 0.05). On the other hand, 1.25% PPH decreased (*P* < 0.05) the relative abundance of *Lachnospiraceae_XPB1014*, and 3.75% PPH decreased (*P* < 0.05) the relative abundance of *Lachnospiraceae_XPB1014* and unclassified *Bacteroidales* (Fig. 7). Above results demonstrated that different level of PPH in the diet could change the composition of fecal microbes at the genus level, which most were related to the intestinal barrier integrity.

### Pearson correlation analysis

Based on the above findings, an analysis was conducted to explore the potential relationships between serum DAO, L-DA, IL -2, IL-6, TNF-α, IL-4, IL-10, IFN-γ and fecal microbe on the genus level (Table 5). Analysis results revealed a negative correlation between IL-2 and *Lachnospiraceae_unclassified* (*P* < 0.05), IL-2 and *Prevotellaceae_UCG-001* (*P* < 0.05), IL-2 and *Ruminococcus* (*P* < 0.05), IL-6 and *Streptococcus* (*P* < 0.01), IL-6 and *Rikenellaceae_RC9_gut_group* (*P* < 0.05), IL-10 and *Bacteroidales_RF16_group_norank* (*P* < 0.05), IL-10 and *Family_XIII_AD3011_group* (*P* < 0.05). Furthermore, there was a positive correlation between IL-2 and *[Eubacterium]_coprostanoligenes_group_norank* (*P* < 0.05), IL-6 and *Paludibacteraceae_unclassified* (*P* < 0.05), IL-6 and *Prevotellaceae_UCG-001* (*P* < 0.05), TNF-α and *Treponema* (*P* < 0.05), IL-10 and *Prevotella* (*P* < 0.01), IL-10 and *Anaerovibrio* (*P* < 0.05), IFN-γ and *Prevotellaceae_uncultured* (*P* < 0.05). However, no significant correlations were found between DAO and fecal microbe (*P* > 0.1), or D-LA and fecal microbe (*P* > 0.1) (The complete Pearson correlation analysis is presented in the additional file 2).

**Table 5:** The Pearson correlation analysis

Item	DAO	D-LA	IL-2	IL-6	TNF-α	IL-4	IL-10	IFN-γ
*Streptococcus*	N	N	N	-0.557**	N	N	N	N
*Rikenellaceae_RC9_gut_group*	N	N	N	-0.418*	N	N	N	N
*Treponema*	N	N	N	N	0.382*	N	N	N
*Prevotella*	N	N	N	N	N	N	0.530**	N
*Prevotellaceae_NK3B31_group*	N	N	N	N	N	N	N	N
*Bacteroidales_RF16_group_norank*	N	N	N	N	N	N	-0.378*	N
*[Eubacterium]_coprostanoligenes_group_norank*	N	N	0.391*	N	N	N	N	N
*Lachnospiraceae_unclassified*	N	N	-0.451*	N	N	N	N	N
*Paludibacteraceae_unclassified*	N	N	N	0.364*	N	N	N	N
*Prevotellaceae_UCG-001*	N	N	-0.408*	0.391*	N	N	N	N
*Ruminococcus*	N	N	-0.426*	N	N	N	N	N
*Prevotellaceae_uncultured*	N	N	N	N	N	N	N	0.363*
*Anaerovibrio*	N	N	N	N	N	N	0.418*	N
*Family_XIII_AD3011_group*	N	N	N	N	N	N	-0.436*	N

* 0.01 < *P* ≤ 0.05, ** 0.001 < *P* ≤ 0.01, N means there is no statistical difference between two indicators, *P* > 0.05. *n* = 6

### The carcass traits and meat quality of growing-finishing pigs

#### The carcass traits and meat characteristics

The carcass traits and meat characteristics of finishing pigs are shown in Table 6. Compared to the control group, dietary 2.5% PPH increased the live weight (*P* < 0.01), hot carcass weight (*P* < 0.01) and back fat depth (*P* < 0.05) of finishing pigs. The carcass length, carcass yield and loin muscle area didn’t show obvious differences. Actually, except for the pH value at the 45th min after slaughter (*P* < 0.05), there was no significant difference in the meat characteristics of LT between two groups (*P* < 0.10).

**Table 6:** Effects of PPH supplement on the carcass traits of pigs and meat characteristics of the LT

Item^1^	Control	2.5% PPH	SEM	*P*- Value
**Carcass traits**				
Live weight, kg	110.2	123.4	3.428	0.003
Hot carcass weight, kg	83.0	94.77	3.127	0.004
Carcass oblique length, cm	93.33	95.17	1.635	0.288
Carcass straight length, cm	102.3	105.2	1.753	0.137
Carcass yield, %	75.32	76.76	0.818	0.108
The backfat thickness, cm	2.267	2.867	0.218	0.020
Loin muscle area, cm^2^	36.99	39.86	4.274	0.359
**Meat characteristics**				
pH 45 min	5.673	5.860	0.083	0.049
pH 24 h	5.167	5.210	0.049	0.399
^1^Lightness, L*	42.65	39.34	0.013	0.096
^2^Redness, a*	7.977	8.653	0.053	0.309
^3^Yellowness, b*	3.217	3.178	0.469	0.935
^4^Pressure loss, %	35.86	37.91	1.644	0.242
^5^Cooking loss, %	19.62	17.38	2.608	0.412
^6^Marbling score	2.500	2.750	0.310	0.438
Shear force of fresh meat, N/cm^2^	7.132	7.600	0.308	0.159
Shear force of cooked meat, N/cm^2^	21.45	21.73	2.354	0.907

^1^L* measures darkness (0) to lightness (100). ^2^a* measures redness (greater a* value indicates a redder color)

^3^b* measures yellowness (greater b* value indicates a yellow color). ^4^Pressure loss = [(initial weight − pressed weight) / initial weight] ×100%. ^5^Cooking loss = [(initial weight of LT sample− cooked weight of LT sample) / initial weight of LT sample] ×100%. ^6^NPPC marbling based on the 1999 standards measured in half-point increments; 1 = least amount of marbling, 6 = most amount of marbling. Data are presented as mean and total SEM, *n* = 6. *P* ≤ 0.05 means there is statistical difference between the control group and 2.5% PPH group

#### The FAAs profile of LT

As shown in the Table 7, 2.5% PPH supplement not only tended to reduce the total FAAs (*P* = 0.092) and free glycine (Gly, *P* = 0.077), but also reduced the free carnosine (Car, *P* = 0.01) in the LT muscle. As for other flavor amino acids or peptides, such as free Glu, Asp, Ala and Ans, no obvious difference was observed between the control group and 2.5% PPH group (*P* > 0.1).

**Table 7:** Effect of PPH supplement on the FAAs of LT

Item^1^, mg/100 g	Control	2.5% PPH	SEM	*P*- Value
P-Ser	1.015	0.908	0.132	0.437
Tau	41.78	42.62	7.090	0.908
Asp	2782	2661	161.7	0.481
Thr	4.699	4.478	0.487	0.660
Ser	4.206	3.858	0.369	0.366
Glu	5.108	5.756	0.484	0.224
Gln	26.78	26.96	3.852	0.963
α-AAA	2.71	2.307	0.470	0.417
Gly	12.37	10.73	0.818	**0.077**
Ala	25.39	24.29	1.734	0.537
Cit	2.324	2.431	0.339	0.760
Val	5.79	5.533	0.422	0.555
Cys	1.688	1.562	0.540	0.821
Met	4.137	3.129	0.729	0.197
Ile	4.125	4.008	0.683	0.868
Leu	5.513	5.689	0.587	0.771
Tyr	4.094	3.877	0.367	0.567
Phe	4.742	4.319	0.350	0.259
β-Ala	5.417	4.396	0.783	0.221
β-AiBA	3.007	2.346	0.431	0.157
Trp	1.398	1.405	0.316	0.982
Lys	6.362	6.184	0.589	0.769
His	2.543	2.831	0.162	0.111
Ans	33.61	30.27	3.488	0.363
Car	1186	997.9	59.63	**0.010**
Arg	4.863	4.234	0.618	0.333
Total FAAs	4185	3862	159.1	**0.092**

P-Ser = Phosphorylated serine; Tau = Taurine; Asp = Aspartic Acid; Thr = Threonine; Ser = Serine; Glu = Glutamic Acid; Gln = Glutamine; α-AAA = α-Aminoadipic acid; Gly = Glycine; Ala = Alanine; Cit = Citrulline; Val = Valine; Cys = Cysteine; Met = Methionine; Ile = Isoleucine; Leu = Leucine; Tyr = Tyrosine; Phe = Phenylalanine; β-Ala = β-Alanine; β-AiBA = β-aminoisobutyric acid; Trp = Tryptophan; Lys = Lysine; His = Histidine; Ans = Anserine; Car = Carnosine; Arg = Arginine; FAAs = Free amino acids

^1^ Values are expressed by means with total SEM, *P* ≤ 0.05 means there is statistical difference between the control group and 2.5% PPH group. *n* = 6

#### The LCFAs profile of LT

In addition, except for the greater ratio of C20:0 (*P* < 0.05) in the LT muscle from 2.5% PPH group, most LCFA in the LT muscle didn’t show remarkable difference between two groups (*P* > 0.1) (Table 8).

**Table 8:** Effect of PPH supplement on the LCFAs profile of LT

Item^1^, %	Control	2.5% PPH	SEM	*P*-Value
C12:0	0.075	0.072	0.002	0.209
C14:0	1.281	1.211	0.105	0.521
C16:0	27.66	27.93	0.326	0.420
C16:1	3.203	2.873	0.394	0.423
C17:0	0.201	0.187	0.021	0.535
C18:0	14.45	15.71	1.216	0.325
C18:1, n-9t	0.137	0.136	0.005	0.851
C18:1, n-9c	40.41	38.76	2.663	0.549
C18:2, n-6c	8.807	9.406	1.536	0.704
C20:0	0.183	0.217	0.014	**0.048**
C20:1	0.746	0.715	0.075	0.684
C18:3, n-3	0.262	0.269	0.017	0.680
C20:2	0.255	0.258	0.023	0.895
C20:3, n-6	0.279	0.305	0.066	0.704
C20:4, n-6	1.933	1.951	0.544	0.973
Saturated fatty acids	43.83	45.32	1.261	0.268
Monounsaturated fatty acids	44.49	42.46	3.102	0.526
Polyunsaturated fatty acids	11.54	12.15	2.114	0.778

^1^ Values are expressed by means with total SEM, *P* ≤ 0.05 means there is statistical difference between the control group and 2.5% PPH group. *n* = 6

## Discussion

Yeast is a single-cell eukaryotic microorganism belonging to fungi, which contains the similar organelles to the eukaryotic cells [23]. There are many evidences that yeasts and yeast-based products are natural growth enhancer in improving nutrient utilization and growth performance of livestock [24–26], which was further verified by PPH in growing-finishing pigs in this study. Dietary brewer’s yeast hydrolysate linearly improved daily gain and gain/feed ratio in finishing pigs when the addition level is less than 1% [26]. Combined with our findings in the greater ADG and ADFI of pigs, we speculated that PPH had obvious linear effect in enhancing the growth performance of pigs fed with less than 2.8%, which may be explained that enzymes contained in yeast, such as phytase, help to improve the utilization of some essential nutrients, including amino acids and minerals [27]. However, the quadratic regression effect of PPH dose indicated that excess amounts didn’t always make sense in increasing the growth rate of pigs, which may be related to the high nucleic acid content or the deficiency of some essential nutrients in the diet when the feed formula is corn-soybean based feed.

Amm is a natural, endogenous molecule generated as a by-product of protein ingestion via the catabolism of amino acids, small nitrogenous components of proteins, largely occurring in the liver and gut [28]. In this study, PPH supplement significantly reduced the serum Amm, suggesting that PPH could reduce the endogenous catabolism of amino acids derived from dietary protein [29], thus supporting greater growth performance of growing-finishing pigs in the PPH group, especially in the 2.5% group and 3.75% group. However, the growing-finishing pigs in the 5% PPH group with the lowest serum Amm didn’t present the highest growth performance among five groups, and the regulation of serum Amm removal in the pigs fed with 5% PPH remain unclear. Besides that, in the absence of infection or renal biomarkers, the greater serum ALB and CREA were usually regarded that both hepatic protein synthesis and muscle metabolism were motivated [30, 31]. Therefore, we speculated that PPH supplemented as a functional protein source could improve the growth performance of pigs via promoting metabolic efficiency, as well as the carcass traits of pigs. Certainly, further direct evidence is needed to be provided to support this point.

Indeed, pork quality is influenced by many factors, such as breed, gender, feed nutrition, rearing environment, and slaughter method [32]. It has been reported that growth rate is positively correlated with the pH value and water content of the pork, and negatively associated with the marbling score and color of meat [33], which was in line with our results about the pH_45min_ of LT. Moreover, accelerated growth rates in pigs compromised the deposition of key nutrients, such as amino acids that affect flavor development [34, 35]. In addition, it has been reported that higher-protein diets increased the concentrations of carnosine and FAAs [36]. In this study, the total FAAs and Car of LT in the 2.5% PPH group was less than that in the CON group, which wasn’t consistent with previous study that an increase in slaughter weight is closely related to enhanced flavor [37]. Thus, the possible reason may be owing to the rapid growth rate of pigs intensify the consuming of amino acids, and subsequently reduce the synthesis of Car. Actually, there was no big difference in the other meat characteristics, indicating that meat quality, including flavor and taste of meat [38], is primarily determined by breed under identical rearing, slaughtering, gender and feed formulation. However, a study on the optimal PPH level for achieving the best meat quality was not conducted, and further research is required to clarify.

Simultaneously, besides protein and amino acids, the micronutrients of the yeast cell components, such as mannans, β-glucans and nucleotides, also played immunomodulatory effects via activating immune responses, including the production of molecules and some immune cells [39, 40]. Dietary yeast hydrolysate could improve growth performance, serum immune cytokine levels, and intestinal probiotic in growing-finishing pigs [41], and improve intestinal morphology and immune status in weaned piglets [12, 42]. Significantly, we found PPH, provided as partial substitution of the soybean proteins, could obviously increase serum antioxidants, immunoglobulin and anti-inflammatory factors, which further demonstrated that PPH supplement activated the antioxidant system and immune response of growing-finishing pigs, especially when the addition dose is between 3.75% and 5%. The immune system is regulated by immune organs, immune cells, soluble cytokines, and so on [43]. It has been demonstrated that pigs fed yeast with a higher anti-inflammatory ability [44, 45]. Similar to the trend of serum immune-related indexes, both blood eosinophils and basophils proportion increased in pigs fed with 5% PPH. However, the optimal dose of PPH for growth-enhancing or immuno-enhancing effect wasn’t completely consistent. According to previous studies, there is a negative relationship between the growth and immunity performances of animals [46, 47]. Based on the levels of serum antioxidant-related indicators, immunoglobulins and serum intestinal permeability markers in this study, the growth performance of pigs showed positive correlation with immunity performances when the addition amount of PPH is no more than 2.5% in the feed. Otherwise, there may be a negative relationship between growth rate and immunity performance in pigs when dietary PPH supplementation exceeds the optimal level. Therefore, whether 3.75% and 5% PPH cause overstimulationof the immune system in growing-finishing pigs, and whether the reduced growth performance observed at these inclusion levels results from such overstimulation, requires further investigation.

It is conceivable that yeast could be supplemented as a probiotic in livestock. In this study, 1.25% and 3.75% PPH supplement lowered the microbiota richness via reducing the ACE and Chao1 indexes, and 2.5% PPH improved microbiota diversity via reducing the Simpson index, which was consistent with previous study that replacement of the 40% soybean protein in weanling piglet diets by yeast help to be enriched with probiotics with lower α diversity in the colon [7], indicating that 2.5% PPH supplement could improve the microbiota diversity without reducing species richness whereas the α diversity of the microbe in the 2.5% PPH also reduced when compared with that in the control group. One point worth emphasizing is that the lower α diversity is associated with greater growth performance in swine [7, 48, 49], which are in line with our results on α diversity of fecal microbiota, final weight and ADG of growing-finishing pigs in the 2.5% and 3.75% groups. These findings further strengthenzed the point that the α diversity of microbiota is associated with the growth performance of pigs.

As expected, the fecal bacterial composition has been changed by 1.25%-5% PPH supplement in the diet of pigs according to the α diversity. It has been shown that the abundance of *Clostridia_UCG-014* is negatively correlated with colitis [50], while the abundance of *Lachnospiraceae_XPB1014* is positively correlated with the inflammation level and lipid metabolism [49], thus indicating that above changes in the relative abundance of *Clostridia_UCG-014* and *Lachnospiraceae_XPB1014* are beneficial to enhancing the intestinal mucosal barrier and reducing the inflammation level of pigs fed with diet including 1.25%, 2.5% and 3.75% PPH, respectively. As an obligate predatory bacterium, *Bradymonadales* is beneficial to the construction of the intestinal microbial community and the recycling of nutrients [51], which means that greater *Bradymonadales* in 1.25% PPH group may improve the stability of the intestinal flora composition or the efficiency of nutrient recycling in pigs. *Prevotellaceae* is considered to be related to the synthesis of short-chain fatty acids, and its increased abundance is beneficial to the protection of the intestinal mucosal barrier [52]. Meanwhile, the abundance of the intestinal pathogenic genus *Bacteroidales* in 3.75% PPH group is lower than that in the control group, inferring that 3.75% and 5% PPH in the diet may contribute to enhancing the intestine homeostasis.

Beyond the microbial composition of the gut and systemic immune factors, the integrity of intestine is paramount for maintaining intestine homeostasis [53, 54]. Serum DAO and D-LA, as well as intestinal morphology serve as typical biomarkers to assess the intestinal integrity and permeability [55, 56]. It has been reported that the lowered DAO activity and D-LA content were contributed to increase the villi height of the small intestine [57, 58], suggesting that the D-LA concentration and DAO activity were negatively correlated with intestinal barrier integrity and health. Additionally, previous study had proved that dietary yeast hydrolysate could decrease the serum DAO in weaned piglets [24], which was consistent with our findings, thus making it a reliable measure for improving the intestinal integrity of pigs via supplementing 2.5%-5% PPH to replace soybean meal in the diet. Unfortunately, the final weight and ADG of pigs did not increase accordingly, although the levels of DAO and D-LA in the 3.75% and 5% PPH groups significantly decreased. To the authors’ best knowledge, it was suspected that excessively low level of serum DAO activity and D-LA concentration would affect the growth rate of pigs for the lower absorption and transport of nutrients in the intestinal tract for the relatively lower intestinal permeability.

D-LA is exclusively produced through bacterial metabolism in the intestines and can provide insights into changes in intestinal permeability [59]. However, no obvious relationship between DAO or D-LA levels and the fecal microbiota at the genus level in this study. On the other side, based on the relationship between serum cytokines and some fecal microbe, we speculated that the levels of *Prevotellaceae_UCG-001*,* Treponema*,* [Eubacterium]_coprostanoligenes_group_norank*, *Paludibacteraceae_unclassified*,* Bacteroidales_RF16_group_norank* and *Family_XIII_AD3011_group* would increase with the degree of inflammation, but *Lachnospiraceae_unclassified*,* Ruminococcus*,* Streptococcus*,* Rikenellaceae_RC9_gut_group*,* Prevotella*,* Anaerovibrio*,* Prevotellaceae_uncultured* would enrich in the colon when the pigs in the state of lower inflammation. Interestingly, *Prevotellaceae_UCG-001* level was positive with serum pro-inflammatory cytokine IL-6, but negatively correlated with serum pro-inflammatory cytokine IL-2. In this study, the *Prevotellaceae_UCG-001* abundance increased in the pigs fed with 5% PPH, which have the highest levels of anti-inflammatory factor and immunoglobulin, thus indicating that *Prevotellaceae_UCG-001* tend to be as a probiotic in the absence of significant changes in the levels of IL-2 and IL-6, which was consistent with previous study [52]. Consequently, we speculated that dietary different level of PPH to the diet of pigs could improve the intestinal microecological environment via increasing the proportion of probiotics, and 3.75% and 5% PPH are more conducive to enhancing the intestinal mucosal barrier of pigs.

## Conclusions

In summary, as a promising alternative protein source, partially replacing soybean meal with PPH in the diet can improve the growth performance, carcass production of pigs, as well as enhancing stress resistance capability and intestinal mucosal barrier function, thereby helping to maintaine intestine homeostasis in growing-finishing pigs. Our findings suggest that a dietary inclusion level of 2.79% PPH may be optimal for achieving the greatest economic benefit. Hence, considering the price gap between PPH and soybean meal, supplementing PPH as functional protein source is a feasible strategy to increase production efficiency and promote a more diversified feed protein system with soybean meal as the foundational protein source.

## Supplementary Information

Below is the link to the electronic supplementary material.

Supplementary Material 1

Supplementary Material 2

## Data Availability

The datasets used or analyzed during the current study are available from the corresponding author on reasonable request.

## Abbreviations

PPH: *Pichia pastoris* hydrolysate
SCP: Single cell protein
TP: Total protein
Amm: Ammonia
ALB: Albumin
CREA: Creatinine
GLU: Glucose
BUN: Blood urea nitrogen
TG: Triglyceride
HDL: High-density lipoprotein
LDL: Low-density lipoprotein
ACE: Abundance-based coverage estimator
IL-4: Interleukin-4
IL-10: Interleukin-10
IFN-γ: Interferon gamma
IgM: Immunoglobulin M
LT: Longissimus thoracis
LCFAs: Long-chain fatty acids
TCHO: Total cholesterol
SOD: Superoxide dismutase
GSH-Px: Glutathione peroxidase
T-AOC: Total antioxidant capacity
MDA: Malondialdehyde
DAO: Diamine oxidase
D-LA: D-lactic acid
NRC: National Research Council
ADG: Average daily gain
ADFI: Average daily feed intake
F/G: Feed to gain
IL-2: Interleukin-2
IL-6: Interleukin-6
TNF-α: Tumor necrosis factor-α
IgG: Immunoglobulin G
IgA: Immunoglobulin A
FAAs: Free amino acids
