# Supplementary material for: The functional evaluation of Pichia pastoris hydrolysate as a protein source partial replacement of soybean meal in diets of growing-finishing pigs
Source: Porcine Health Manag. 2026 Apr 17;12:29. doi: 10.1186/s40813-026-00511-7 (PMC13127005; doi:10.1186/s40813-026-00511-7)
Supplement: Supplementary file 1 — Supplementary Material 1 [file 40813_2026_511_MOESM1_ESM.docx]

Table 3 Effect of PPH supplement on the routine blood parameters of pigs

| Item | 0 | 1.25% | 2.5% | 3.75% | 5% | SEM | *P-Value* |
| --- | --- | --- | --- | --- | --- | --- | --- |
| WBC, 10^9^/L | 18.26 | 19.51 | 19.47 | 15.60 | 21.56 | 1.342 | 0.742 |
| Neutrophils, 10^9^/L | 1.867ab | 1.935ab | 1.707b | 1.563b | 3.017a | 0.197 | 0.135 |
| Lymphocytes, 10^9^/L | 16.14 | 17.31 | 17.49 | 13.81 | 18.03 | 1.219 | 0.844 |
| Monocytes, 10^9^/L | 0.178 | 0.138 | 0.183 | 0.118 | 0.212 | 0.018 | 0.481 |
| Eosinophils, 10^9^/L | 0.078b | 0.115b | 0.087b | 0.088b | 0.228a | 0.019 | 0.062 |
| Basophils, 10^9^/L | 0.002b | 0.017ab | 0.000b | 0.017ab | 0.067a | 0.009 | 0.147 |
| Neutrophils, % | 9.955 | 9.560 | 8.835 | 13.617 | 14.433 | 1.088 | 0.369 |
| Lymphocytes, % | 88.62 | 89.14 | 89.75 | 84.12 | 83.33 | 1.304 | 0.384 |
| Monocytes, % | 0.958 | 0.718 | 0.965 | 1.483 | 0.933 | 0.179 | 0.764 |
| Eosinophils, % | 0.452b | 0.532ab | 0.447b | 0.700ab | 1.083a | 0.092 | 0.145 |
| Basophils, % | 0.017b | 0.050ab | 0.000b | 0.083ab | 0.217a | 0.027 | 0.074 |
| Red blood cell count, 10^12^/L | 6.685a | 6.708a | 7.435a | 4.277b | 7.052a | 0.363 | 0.038 |
| Hemoglobin, g/L | 116.3ab | 117.5ab | 134.8a | 79.0b | 126.2a | 6.349 | 0.046 |
| Hematocrit, % | 40.65a | 41.05a | 45.92a | 26.35b | 42.72b | 2.214 | 0.04 |
| Mean corpuscular volume, fL | 60.78 | 61.62 | 61.95 | 61.35 | 60.43 | 0.483 | 0.879 |
| Mean corpuscular hemoglobin, pg | 17.38b | 17.72ab | 18.20ab | 18.67a | 17.85ab | 0.182 | 0.216 |
| Mean corpuscular hemoglobin concentration, g/L | 286.0b | 287.7b | 294.0ab | 304.5a | 295.7ab | 2.084 | 0.028 |
| Platelet count, 10^9^/L | 107.2 | 194.5 | 196.0 | 174.2 | 174.3 | 21.03 | 0.695 |
| Mean platelet volume, fL | 9.55 | 9.30 | 9.42 | 9.03 | 9.95 | 0.191 | 0.674 |
| Plateletcrit, % | 0.101 | 0.193 | 0.185 | 0.172 | 0.178 | 0.021 | 0.674 |
